# Development of ADS051, an oral, gut‐restricted, small molecule neutrophil modulator for the treatment of neutrophil‐mediated inflammatory diseases

**DOI:** 10.1002/2211-5463.13668

**Published:** 2023-07-10

**Authors:** Christopher K. Murphy, Bharat Dixit, Frederick B. Oleson, Roland E. Dolle, Ronald Farquhar, Beth A. McCormick

**Affiliations:** ^1^ Adiso Therapeutics, Inc. (formerly Bacainn Therapeutics, Inc.) Concord MA USA; ^2^ Independent Consultant Carlisle MA USA; ^3^ Medicine Inventions LLC Eureka MO USA; ^4^ Department of Microbiology and Physiological Systems University of Massachusetts Chan Medical School Worcester MA USA; ^5^ Program in Microbiome Dynamics University of Massachusetts Chan Medical School Worcester MA USA; ^6^ Present address: Resolvix Bio, Inc Boston MA USA

**Keywords:** ADS051, cyclosporine A, formyl peptide receptor 1, multidrug resistance protein 2, neutrophil transmigration, small molecule

## Abstract

Neutrophils are an essential component of the innate immune system; however, uncontrolled neutrophil activity can lead to inflammation and tissue damage in acute and chronic diseases. Despite inclusion of neutrophil presence and activity in clinical evaluations of inflammatory diseases, the neutrophil has been an overlooked therapeutic target. The goal of this program was to design a small molecule regulator of neutrophil trafficking and activity that fulfilled the following criteria: (a) modulates neutrophil epithelial transmigration and activation, (b) lacks systemic exposure, (c) preserves protective host immunity, and (d) is administered orally. The result of this discovery program was ADS051 (also known as BT051), a low permeability, small molecule modulator of neutrophil trafficking and activity via blockade of multidrug resistance protein 2 (MRP2)‐ and formyl peptide receptor 1 (FPR1)‐mediated mechanisms. ADS051, based on a modified scaffold derived from cyclosporine A (CsA), was designed to have reduced affinity for calcineurin with low cell permeability and, thus, a greatly reduced ability to inhibit T‐cell function. In cell‐based assays, ADS051 did not inhibit cytokine secretion from activated human T cells. Furthermore, in preclinical models, ADS051 showed limited systemic absorption (<1% of total dose) after oral administration, and assessment of ADS051 in human, cell‐based systems demonstrated inhibition of neutrophil epithelial transmigration. In addition, preclinical toxicology studies in rats and monkeys receiving daily oral doses of ADS051 for 28 days did not reveal safety risks or ADS051‐related toxicity. Our results to date support the clinical development of ADS051 in patients with neutrophil‐mediated inflammatory diseases.

## Graphical abstract

Uncontrolled neutrophil activity can lead to inflammation and tissue damage in acute and chronic diseases. Approved small molecules targeting neutrophils display efficacy and safety limitations. ADS051 is an orally administered, gut‐restricted, novel small molecule modulator of neutrophil trafficking and activity via blockade of MRP 2‐ and FPR1‐mediated mechanisms. Our results support clinical development of ADS051 for treating neutrophil‐mediated diseases.

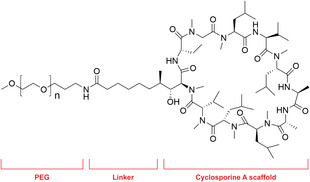

**Abbreviations**
CsA: cyclosporine A
ECGs: electrocardiograms
fMLF: formylated Met‐Leu‐Phe
FPR1: formyl peptide receptor 1
GI: gastrointestinal
HEPES: N‐2‐hydroxyethylpiperazine‐N′‐2‐ethanesulfonic acid
HXA_3_
: hepoxilin A_3_
JAK: janus kinase
MDR1: multidrug resistance 1
MeBmt: (4*R*)‐4‐[(*E*)‐2‐butenyl]‐4,*N*‐dimethyl‐l‐threonine
MRP2: multidrug resistance protein 2
NOEL: no observed effect level
PBMC: peripheral blood mononuclear cells
PEG: polyethylene glycol
Pen/Strep: penicillin/streptomycin
RA: rheumatoid arthritis
ROS: reactive oxygen species
S1P: sphingosine‐1‐phosphate
SLE: systemic lupus erythematosus
TK: toxicokinetic
UC: ulcerative colitis

Neutrophils, essential to the innate immune system, are key immunological first responders in unhealthy human tissues. They migrate to sites of inflammation and infection, where they become activated releasing destructive molecules and proteins, including toxic reactive oxygen species (ROS) and neutrophil extracellular traps, to clear pathogens [1, 2, 3]. However, uncontrolled neutrophil activity can lead to inflammation and tissue damage in chronic diseases, including asthma and other pulmonary diseases, rheumatoid arthritis (RA), diabetes, and ulcerative colitis (UC) [1, 2, 3, 4, 5]. This has inspired the development of therapeutics targeting neutrophils, including small molecules. Novel small molecules have the advantage over biologics of oral administration and, among those targeting neutrophils, include a sphingosine‐1‐phosphate (S1P) receptor modulator (ozanimod) and a Janus kinase (JAK) inhibitor (tofacitinib) [2, 6, 7, 8, 9]. Ozanimod is FDA‐approved for moderately to severely active UC [8]. Tofacitinib is FDA‐approved for the treatment of RA, UC, and psoriatic arthritis [2, 7, 10]. Multiple clinical trials have investigated neutrophil‐targeting agents, including tofacitinib, in the context of several diseases and have found that treatment side effects exist [2, 7, 8]. Consequently, in the case of UC, current effective treatment options, including tofacitinib and upadacitinib, have boxed warnings in the United States related to increased risk of malignancies, infections, major adverse cardiovascular events, and thrombosis [11, 12]. Furthermore, many patients do not respond to treatments, with loss of response over time an additional concern [7, 13]. Therefore, due to the current therapies' efficacy and safety limitations, there is a considerable need for better, more selective treatment options for multiple diseases whose pathophysiologies involve neutrophils.

In inflammation‐driven diseases such as UC, uncontrolled or inappropriate neutrophil activity damages tissue [14, 15, 16]. Neutrophil infiltration into the colon is a hallmark of UC, with the extent of neutrophilic infiltration directly proportional to the degree of mucosal lining epithelial cell necrosis and, thus, the severity of the disease [1, 16]. For this reason, neutrophil fecal calprotectin is a routinely used and validated biomarker for assessing UC activity in the clinic [16, 17]. The presence of neutrophils in the colonic mucosa has been correlated with increased patient hospitalization and corticosteroid use [14]. In addition, biopsies from patients with UC show higher expressions of NET‐associated proteins [15]. In other diseases with a high proportion of neutrophils, including asthma, chronic obstructive pulmonary disease, and bronchiectasis, it has been shown that neutrophils cause tissue damage and severe disease [4]. High numbers of neutrophils and their uncontrolled cytotoxicity from release of ROS and proteases cause host tissue damage and destruction in RA and systemic lupus erythematosus (SLE) [5]. The clear evidence of neutrophil involvement in the pathophysiology of many inflammatory disorders makes neutrophils novel and attractive targets for treating inflammatory diseases [5, 10, 18].

Key mechanisms in the late stages of neutrophil migration to, and activation at, sites of inflammation have been elucidated, particularly in the context of the colonic, respiratory, and reproductive epithelial barriers [19, 20, 21, 22, 23, 24, 25, 26, 27, 28]. It has been demonstrated in a human cell‐based system that hepoxilin A_3_ (HXA_3_), an intermediate in the arachadonic acid pathway and a potent neutrophil chemo‐attractant, is effluxed from epithelial cell monolayers by the apically situated multidrug resistance protein 2 (MRP2) in response to pathogen interaction [29, 30]. The resultant HXA_3_ gradient is sensed by human neutrophils added to the basolateral space. Next, the neutrophils transit through the epithelial tight junctions into the apical space (corresponding to the luminal side of the gut epithelium) [29, 30]. Additional evidence for MRP2 involvement in inflammation has been demonstrated in preclinical UC models and from its upregulation in colonic biopsy samples from patients with Crohn's disease and UC [31].

Neutrophils are activated by the binding of formylated peptides to their cell‐surface expressed G‐protein‐coupled receptor, the formyl peptide receptor 1 (FPR1) [20, 32]. Formylated peptides are derived from resident bacteria and mitochondria released from damaged tissue [32]. In preclinical models, blocking FPR1‐formylated peptide interaction inhibited neutrophil activation [33]. The association of neutrophils with inflammatory diseases and the involvement of the MRP2/HXA_3_‐ and FPR1‐mediated trafficking and activation of neutrophils makes these two mechanisms attractive targets for new therapies [29, 30].

We designed ADS051 (also known as BT051) to be an orally administered, gut‐restricted, selective neutrophil activity modulator that blocks neutrophil trafficking into and activation within the colon via MRP2‐ and FPR1‐mediated mechanisms. Here, we report the results of the ADS051 preclinical program, including its mechanisms of action, pharmacokinetics, and safety characterizations. Our results support the continued clinical development of ADS051 for treating neutrophil‐mediated diseases.

## Results

### ADS051 characteristics

**Fig. 1:**
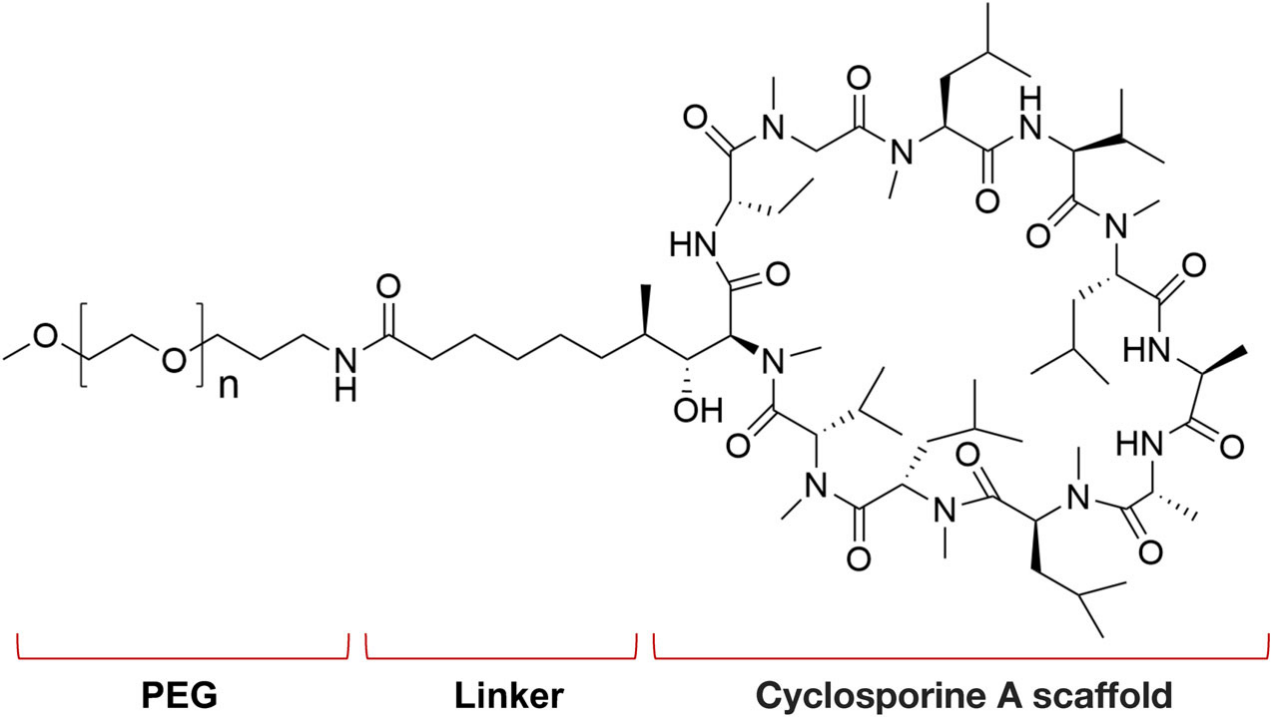
Structural formula of ADS051. Molecular Formula: C170H260N12O48; MW: ~3244 Da (due to variability in the PEG MW, ADS051 can be ± 400 Da of this MW).

ADS051 consists of a cyclosporine A (CsA) scaffold covalently attached via an amide linker to 2000 g·mol^−1^ molecular weight (MW) polyethylene glycol (PEG) (Fig. 1). CsA was chosen as the starting molecular scaffold for ADS051 principally due to its known ability to inhibit MRP2/HXA_3_‐ and FPR1‐mediated neutrophil epithelial transmigration and activation [31, 34, 35, 36]. We sought to eliminate the T‐cell suppressive activity of the CsA scaffold yet retain the inhibition of the two pathways of interest. The amide linker on CsA at position 1 replaces the natural (4*R*)‐4‐[(*E*)‐2‐butenyl]‐4,*N*‐dimethyl‐l‐threonine (MeBmt) residue of CsA. The position of the amide linker was chosen based on the finding that modifications of that residue in CsA significantly reduced calcineurin inhibitory activity [37, 38]. It is widely known that blocking the calcineurin‐cyclophilin complex reduces T‐cell activation and the resulting cytokine expression [39]. The ADS051 rational design also ensures that, unlike CsA, it exhibits limited systemic exposure by its covalent PEG modification while acting locally in the gut to inhibit neutrophil epithelial transmigration and activation.

**Fig. 2:**
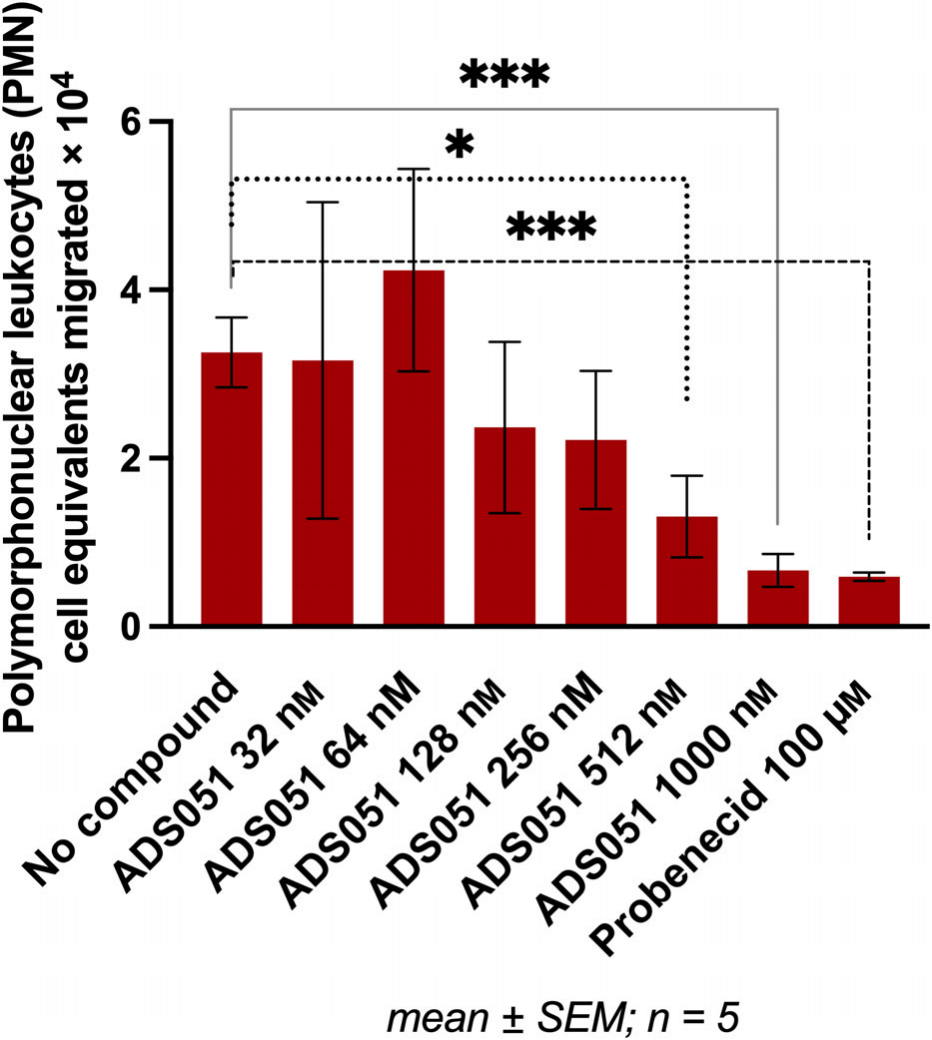
Mean neutrophil migration inhibition in the HXA_3_/MRP2‐driven neutrophil migration/activation assay by ADS051 and probenecid. Human polymorphonuclear cell (neutrophil) transmigration assays, utilizing a T84 human intestinal epithelial cell line, were performed to assess the *in vitro* efficacy of ADS051. Following stimulation via pathogenic *S. typhimurium* to elicit HXA_3_ efflux, migration of fresh human neutrophils from the basolateral side of the polarized monolayer (in response to the HXA_3_ gradient) to the apical side was quantified by MPO activity assay. Statistical analysis was performed using graphpad prism's unpaired, 2‐tailed *t*‐test. ADS051 concentration range: 32 nm–1 μm; Probenecid (positive control) concentration: 100 μm; Statistical significance, when present, noted in graph. *** extremely significant (*P* = 0.0005 for 1 μm ADS051 and 0.0008 for 100 μm probenecid); * significant (*P* = 0.0160 for 512 nm ADS051). (graphpad prism formatting style for *P* values: *P < 0.05, ***P < 0.001).

In our experiments, ADS051 produced dose‐responsive inhibition of inflammation‐induced (HXA_3_/MRP2‐driven) neutrophil epithelial transmigration in human polymorphonuclear cell (neutrophil) *in vitro* assays. As described under Materials and Methods, in the neutrophil transmigration assay, a polarized human intestinal cell line monolayer was grown on a transwell insert to mimic the intestinal epithelial barrier. The cells were then apically stimulated by the addition of pathogenic *Salmonella typhimurium*, eliciting HXA_3_ efflux via MRP2 into the apical side of the chamber. Fresh, unstimulated human neutrophils were added to the basolateral side of the monolayer, where they detected the HXA_3_ gradient and migrated through the epithelial monolayer to the apical side. They were then quantified by assay of myeloperoxidase (MPO), a neutrophil biomarker. Six different concentrations of ADS051 were tested during the initial dose–response screen (Fig. 2). At its highest concentration (1000 nm), the effect of ADS051 was similar to that of the positive control (probenecid at 100 μm) when added to the apical surface of the *S. typhimurium*‐stimulated epithelial cell monolayer (Fig. 2). This control concentration was chosen because probenecid effectively inhibits HXA_3_ release at 100 μm [31].

**Fig. 3:**
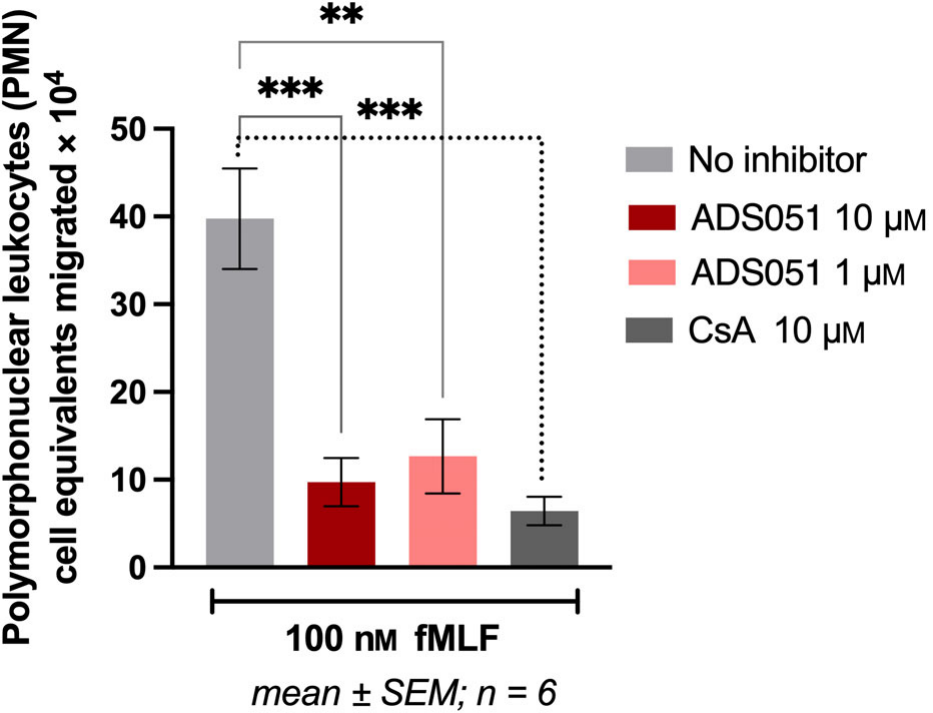
Mean neutrophil migration inhibition in the fMLF‐driven neutrophil transmigration assay by ADS051 and CsA. Fresh human neutrophils were incubated with ADS051 or control media for 1 h at 37 °C and added to the basolateral side of a T84 unstimulated human intestinal epithelial cell monolayer system. A potent neutrophil chemo‐attractant and activator, fMLF, was added to the apical side of the Transwell at 100 nm. The fMLF‐mediated neutrophil migration and activation, mediated by the formyl peptide receptor FPR1, were measured by an MPO activity assay. Statistical analysis was performed using graphpad prism's unpaired, 2‐tailed *t*‐test. PMN = Polymorphonuclear cells (neutrophils); CsA (positive control). No statistically significant change was observed between CsA and either concentration of ADS051. *** extremely significant (*P* = 0.0007 for 10 μm ADS051 and 0.0002 for 10 μm CsA); ** very significant (*P* = 0.0037 for 1 μm ADS051). (GraphPad Prism formatting style for *P* values: **P < 0.01, ***P < 0.001).

ADS051 demonstrated a significant block of formylated Met‐Leu‐Phe (fMLF)‐FPR1 interaction‐mediated neutrophil migration and activation (Fig. 3). A representative ADS051 concentration from the initial dose–response screen described above, specifically 1 μm (1000 nm), as well as 10 μm ADS051 prevented neutrophil transmigration/activation in response to 100 nm fMLF stimulation similarly to 10 μm CsA (Fig. 3).

In summary, ADS051 inhibited the epithelial transmigration and activation of neutrophils in a concentration‐dependent manner in the MRP2/HXA_3_‐ and FPR1/fMLF‐driven assays, similar to the well‐established controls, probenecid, and CsA, respectively (Figs 2 and 3).

In an assay complementary to the HXA_3_/MRP2‐driven neutrophil epithelial transmigration assay, ADS051 was evaluated for activity using Caco‐2 cell monolayers expressing MRP2 or multidrug resistance protein 1 (MDR1), as shown in Fig. S1. Permeability assessments, performed in triplicates [coefficient of variation (CV) < 25%], showed that even at a 25 μm concentration, far below its expected colonic levels, ADS051 effectively inhibited the efflux of the MRP2 substrate, saquinavir, and the MDR1 substrate, talinolol, similarly to control inhibitors (Fig. S1). In addition, we determined that ADS051 was a low permeability compound using MDCKII bidirectional assays with P_app_ values comparable to that of a low permeability control (Lucifer yellow) (Table 1).

**Table 1:** Bidirectional permeability and ER values for ADS051 and controls across cell monolayers. Data are expressed as mean (*n* = 3) ± SD. A‐B, apical to basolateral direction; B‐A, basolateral to apical direction; ER, efflux ratio; ERM, efflux ratio in mock transfected MDCKII cell monolayers; ERT, efflux ratio in transporter expressing MDCKII cell monolayers; LY, Lucifer yellow; NA, not applicable; ND, not determined; P_app_, apparent permeability coefficient; TA, test article.

Compound	MDCKII‐mock‐LV cells			
	Concentration	P_app A‐B_ (× 10^−6^ cm·s^−1^)	P_app B‐A_ (× 10^−6^ cm·s^−1^)	ER_M_
ADS051	14 mg·mL^−1^	0.20 ± 0.04	0.15 ± 0.01	0.73 ± 0.14
	+ Ko143 (1 μm)	0.00 ± 0.00	0.18 ± 0.01	NA
	1.4 mg·mL^−1^	0.43 ± 0.04	0.28 ± 0.01	0.66 ± 0.06
	+ Ko143 (1 μm)	0.49 ± 0.06	0.33 ± 0.07	0.67 ± 0.16
	0.14 mg·mL^−1^	0.00 ± 0.00	0.00 ± 0.00	NA
	+ Ko143 (1 μm)	2.22 ± 0.02	1.12 ± 0.01	0.50 ± 0.01
	0.014 mg·mL^−1^	0.00 ± 0.00	0.57 ± 0.01	NA
	+ Ko143 (1 μm)	0.00 ± 0.00	0.62 ± 0.03	NA
LY	40 μg·mL^−1^	0.20 ± 0.03	NA	ND
LY + ADS051 (TA applied apically)	40 μg·mL^−1^	0.32 ± 0.09	NA	ND
	+ 14 mg·mL^−1^			
LY + ADS051 (TA applied basolaterally)	40 μg·mL^−1^	0.46 ± 0.09	NA	ND
	+ 14 mg·mL^−1^			
Prazosin	1 μm	14.68 ± 13.33	32.01 ± 2.81	2.18 ± 1.99
Prazosin + Ko143	1 μm + 1 μm	33.16 ± 10.22	33.96 ± 2.78	1.02 ± 0.33

Compound	MDCKII‐BCRP‐LV cells				Net‐ER
	Concentration	P_app A‐B_ (× 10^−6^ cm·s^−1^)	P_app B‐A_ (× 10^−6^ cm·s^−1^)	ER_T_	ER_T_‐ER_M_
ADS051	14 mg mL^−1^	0.18 ± 0.01	0.11 ± 0.00	0.63 ± 0.04	−0.10 ± 0.17
	+ Ko143 (1 μm)	0.00 ± 0.00	0.11 ± 0.02	NA	NA
	1.4 mg mL^−1^	0.38 ± 0.01	0.21 ± 0.01	0.56 ± 0.03	−0.11 ± 0.09
	+ Ko143 (1 μm)	0.42 ± 0.02	0.23 ± 0.01	0.54 ± 0.04	−0.14 ± 0.20
	0.14 mg mL^−1^	0.00 ± 0.00	0.00 ± 0.00	NA	NA
	+ Ko143 (1 μm)	0.00 ± 0.00	0.00 ± 0.00	NA	NA
	0.014 mg mL^−1^	0.00 ± 0.00	0.59 ± 0.02	NA	NA
	+ Ko143 (1 μm)	0.00 ± 0.00	0.00 ± 0.00	NA	NA
LY	40 μg mL^−1^	0.06 ± 0.01	NA	ND	ND
LY + ADS051 (TA applied apically)	40 μg mL^−1^	0.21 ± 0.02	NA	ND	ND
	+ 14 mg mL^−1^				
LY + ADS051 (TA applied basolaterally)	40 μg mL^−1^	0.19 ± 0.01	NA	ND	ND
	+ 14 mg mL^−1^				
Prazosin	1 μm	3.02 ± 0.84	53.09 ± 8.15	17.57 ± 5.57	15.39 ± 7.78
Prazosin + Ko143	1 μm + 1 μm	35.50 ± 11.14	31.88 ± 2.00	0.90 ± 0.29	−0.13 ± 0.40

### Secondary pharmacodynamics studies

**Fig. 4:**
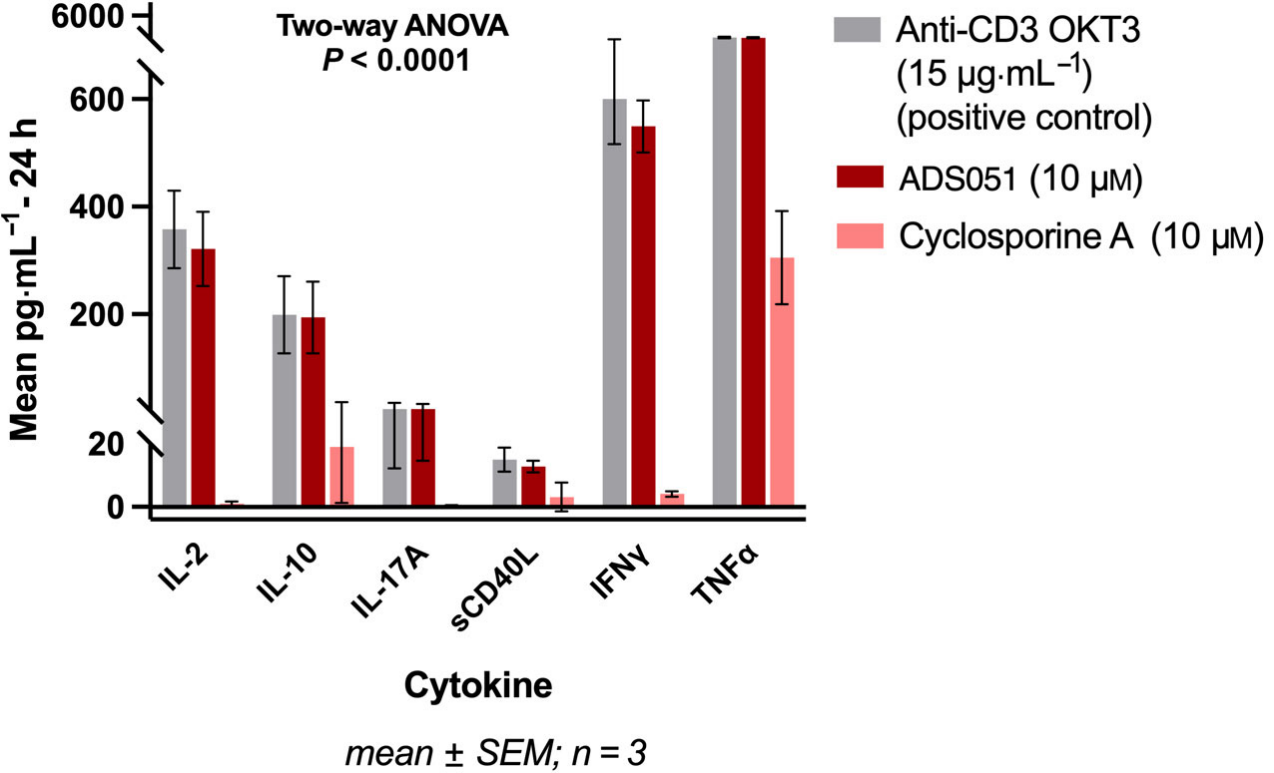
ADS051 does not inhibit cytokine secretion from activated human T cells. T‐cell activation/inhibition assays were performed using human PBMC from 3 healthy human donors to assess T‐cell immunomodulatory activity. The cells (1 × 10^5^ per well), seeded into 96‐well plates, were incubated at 37 °C, 5% CO_2_ for 1 h prior to adding test compounds or controls (CsA or culture medium alone). Then, the test compounds or controls were added and incubated at 37 °C, 5% CO_2_ for 1 h. Anti‐CD3 antibody clone, OKT3, was added to initiate T‐cell activation, and the cultures were incubated at 37 °C, 5% CO_2_ for 24 h. Cytokine/chemokine levels in each sample were determined using the Luminex methodology. Statistical analysis was performed using graphpad prism's 2‐way ANOVA test (*P* < 0.0001).

To determine whether ADS051 had the potential to affect systemic immunosuppression, T‐cell activation/inhibition assays were performed (Fig. 4). A very high concentration of ADS051, namely 10 μm, compared with the expected systemic exposure where T‐cell suppression would be relevant, was tested to determine whether effects at these high levels could be observed (Fig. 4).

ADS051 (10 μm) did not inhibit cytokine secretion from T cells following stimulation with an anti‐CD3 (clone OKT3) antibody, in contrast to the same CsA concentration (Fig. 4). The levels of cytokine elicitation in human peripheral blood mononuclear cells (PBMC) incubated with ADS051 were very similar to those of the positive control anti‐CD3 (clone OKT3) with no added ADS051 at both 24 (Fig. 4) and 48 h (Fig. S2). We included cytokines (among them IL‐2, IFNγ, and TNFα), whose expression is inhibited by CsA [40] and which are critical for T‐cell functioning [41]. Furthermore, we included sCD40L, a cytokine that, upon interaction with CD40, can trigger the synthesis of TNFα and several ILs [42]. The T‐cell activation assessment revealed a statistically significant lack of T‐cell inhibition by ADS051 at 10 μm compared with the potent inhibition exhibited by CsA (Fig. 4).

### Safety pharmacology: gastrointestinal motility assessment following oral administration of ADS051 to Sprague–Dawley rats

To evaluate the effects of ADS051 on gastrointestinal (GI) motility when administered at 0, 50, 300, and 1000 mg·kg^−1^ as a single dose via oral gavage, six male Crl:CD(SD) Sprague–Dawley rats per group were used as described under Materials and Methods. Briefly, rats received a single dose of ADS051 via oral gavage, followed by a charcoal suspension administration via oral gavage 30 min after the test article or the vehicle dose. The test article and charcoal suspension dose volumes were 10 mL·kg^−1^. Thirty minutes after charcoal suspension, administration animals were sacrificed, their intestines removed, and intestinal charcoal transit distance (calculated percent distance traveled and normalized percent inhibition) was evaluated.

A single oral gavage administration of ADS051 at 50, 300, and 1000 mg·kg^−1^ to male Sprague–Dawley rats resulted in no statistically significant alterations in GI motility with a no observed effect level (NOEL) of 1000 mg·kg^−1^ (highest dose tested), as measured by intestinal charcoal transit distance (Table 2). No mortality or abnormal clinical observations occurred during study conduct.

**Table 2:** Internal charcoal transit distance. SD, standard deviation.

Test article	Dose level (mg·kg^−1^)	Intestinal charcoal transit distance ± SD (%)
Vehicle	0	71.5 ± 5.26
ADS051 (Low dose)	50	70.9 ± 5.41
ADS051 (Mid dose)	300	71.4 ± 2.24
ADS051 (High dose)	1000	67.9 ± 6.87

Finally, ADS051 did not produce any neurological or respiratory side effects in Sprague–Dawley rats and did not impact cardiovascular function of the cynomolgus monkeys.

### Definitive toxicology

The objectives of the GLP toxicology studies were to determine the potential toxicity of ADS051 when given orally once daily for 28 consecutive days to Sprague–Dawley rats and cynomolgus monkeys and to evaluate the potential reversibility of any findings over a 28‐day recovery period. The following parameters and end points were analyzed in this study: clinical signs, body weights, body weight gains, ophthalmology, electrocardiology, clinical pathology parameters (hematology, coagulation, clinical chemistry, and urinalysis), gross necropsy findings, organ weights, and histopathologic examinations. Additionally, the toxicokinetic (TK) characteristics of ADS051 were determined in whole blood (Table 3) and its fecal concentrations (Table 4).

**Table 3:** ADS051 whole blood PK parameters on Days 1 and 28 following oral administration of different ADS051 doses in cynomolgus monkeys. AUC_last_, area under concentration time curve from time 0 to last measurement; *C*_max_, maximum concentration; F, female; M, male; NC, not calculable; *t*_1/2_, terminal half‐life; *t*_max_, time to maximum concentration.

Dose	50 mg·kg^−1^·day^−1^		300 mg·kg^−1^·day^−1^		1000 mg·kg^−1^·day^−1^	
Sex of Animals/Number	M	F	M	F	M	F
	3	3	3	3	5	5
Day 1						
AUC_last_ (h × ng·mL^−1^)	127	159	318	2710	1030	1960
*C*_max_ (ng·mL^−1^)	34.1	42.8	95.8	414	191	241
*t*_1/2_ (hour)	1.97	NC	2.24	7.83	NC	7.63
*t*_max_ (hour)	1.00	1.00	0.500	2.00	0.900	3.00
Day 28						
AUC_last_ (h × ng·mL^−1^)	362	382	830	1390	1990	1930
*C*_max_ (ng·mL^−1^)	55.0	46.3	139	282	1110	855
*t*_1/2_ (hour)	NC	NC	NC	NC	7.45	NC
*t*_max_ (hour)	1.17	9.67	0.750	0.833	0.350	1.05

**Table 4:** Average fecal concentrations per dose group in monkeys following first and last dose of ADS051.

Dose (mg·kg^−1^)	50	300	1000
Day 1 Average [ADS051] (μg·g^−1^)	1646	7554	28,518
Day 1 Range [ADS051] (μg·g^−1^)	560 to 2450	5456 to 12 275	6860 to 60 750
Day 24 Average [ADS051] (μg·g^−1^)	3475	12 508	38 779
Day 24 Range [ADS051] (μg·g^−1^)	1904 to 10 420	71 440 to 23 500	9825 to 75 900

There were no ADS051‐related effects at the end of the dosing phase or the recovery phase on mortality, cage‐side observations, detailed clinical observations (including fecal consistency), body weight and body weight gains, food consumption, ophthalmic examinations, electrocardiograms (ECGs), hematology, coagulation, clinical chemistry, urinalysis, or organ weight parameters. Overall, no consistent sex‐related differences in exposure to ADS051 were noted.

Toxicokinetic analysis of ADS051 in the whole blood of dosed monkeys (Table 3) demonstrated that systemic absorption was low. At 50 mg·kg^−1^ in monkeys, the *C*_max_ whole‐blood values ranged from 34 to 55 ng·mL^−1^ (Table 3). The day 1 *C*_max_ blood values for the mid and high doses (300, 1000 mg·kg^−1^) were similar (ranging from 96 to 414 ng·mL^−1^). The day 28 *C*_max_ values at the high dose were four‐ to fivefold higher than those of the mid‐dose (Table 3). Repeat oral administration of ADS051 for 28 consecutive days to male and female rats and monkeys was well‐tolerated at doses up to 1000 mg·kg^−1^·day^−1^. Therefore, the NOEL was considered to be 1000 mg·kg^−1^·day^−1^. At the NOEL, the mean systemic ADS051 *C*_max_ and AUC values for males were 1110 ng·mL^−1^ and 1990 h × ng·mL^−1^, and for females 855 ng·mL^−1^ and 1930 h × ng·mL^−1^, respectively, after 4 weeks of treatment (Table 3).

The amount of ADS051 recovered in feces over the collection period (0–24 h) on Days 1 and 24 generally increased as the dose level increased (Table 4). The majority of ADS051 was recovered during the 8–24 h collection period. After the first dose in the 28‐day studies, ADS051 fecal concentrations were 15 000‐ to 70 000‐fold higher than whole blood *C*_max_ levels in the monkeys (Tables 3 and 4). On Day 1, fecal recovery of ADS051 over a 24 h period post dose in the monkeys was 13% to 38% of the total oral dose administered, and on Day 24, the fecal recovery of ADS051 was 24% to 85% of the total oral dose. Fecal collection beyond 24 h after oral dose administration on Day 1 was not conducted because the animals received a subsequent dose of ADS051 after each 24 h collection period. Not collecting fecal samples beyond 24 h precluded accurate determination of total recovery of dosed ADS051 in the feces. Despite this, the average fecal concentrations over the 24 h periods after the first‐day or last‐day doses greatly exceeded (up to 3000‐fold at the low dose and up to 20 000‐fold at the high dose) the inhibitory levels demonstrated in the neutrophil epithelial transmigration and activation assays with IC_50_s of 1 μm (3.2 μg·mL^−1^) (Table 4).

## Discussion

Uncontrolled neutrophil activity is a key factor in the pathogenesis and clinical course of many inflammatory disorders affecting the digestive, respiratory, and reproductive systems [1, 4, 5, 21, 22, 23, 24, 25, 26, 27, 28]. Clinical development of ADS051 in neutrophil‐mediated disorders, particularly those of the GI tract, is supported by its ability to modulate neutrophil trafficking and activation and potential for improved safety compared with current therapies due to its lack of T‐cell immunosuppression coupled with its lack of significant systemic exposure when administered orally. In *in vitro* efficacy experiments, ADS051 inhibited epithelial transmigration and activation of neutrophils in a concentration‐dependent manner in both the (epithelial cell) HXA_3_/MRP2‐driven assay (Fig. 2) and the (neutrophil) fMLF‐driven (Fig. 3) pathway. The effect on MRP2 was corroborated by its efflux inhibition of the MRP2 substrate, saquinavir, in Caco‐2 cells (Fig. S1). The finding that inhibition was effective at 25 μm ADS051 concentration (Fig. S1), very low compared with its expected colonic levels, further reinforces the biological relevance of its inhibitory effect. In summary, these findings form the current basis of the ADS051 proposed mechanisms of action, and future studies will look at the effect of ADS051 on neutrophils *in vivo*.

Cross talk between neutrophils and T cells is becoming recognized as an important means of coordination between the innate and adaptive arms of the immune system [43, 44]. With the epithelial cell (MRP2)‐directed and neutrophil (FPR1)‐directed activities of ADS051 and its consequential modulation of neutrophil trafficking and activation, the possibility exists that the compound also indirectly reduces T‐cell infiltration and activation within specific tissues. Studies are underway to explore the full functional impact of ADS051, given its direct effects on neutrophils and their modulation of adaptive immune cells.

Our safety pharmacology studies found no ADS051‐related changes in the rat GI, neurological, and respiratory functions, nor any cardiovascular changes in monkeys. GI motility assays were performed as part of safety pharmacology considering that ADS051 contains PEG, and, theoretically, could have unintended additional effects, including diarrhea similar to MiraLAX (PEG 3350) [45]. Importantly, a single administration of ADS051 via oral gavage to Sprague–Dawley rats at dose levels of 50, 300, and 1000 mg·kg^−1^ resulted in no effects on GI motility (Table 2) with a NOEL of 1000 mg·kg^−1^ (highest dose tested). In addition, no abnormalities were observed in monkey GI function or stools during the 28 days of dosing in the toxicology study.

The rats and monkeys receiving 28 days of daily, single, oral doses of ADS051 exhibited no toxicological findings up to the highest doses tested (1000 mg·kg^−1^·day^−1^). These results demonstrate that ADS051 was safe and well‐tolerated, with no dose‐limiting or organ‐specific toxicities. Additionally, there was a significant amount of ADS051 excreted in feces. We will incorporate a more systematic stool collection to fully characterize the elimination profile of ADS051 in future studies.

Toxicokinetic analysis of ADS051 exposure in the whole blood of rats and monkeys revealed that systemic exposure was relatively low. This finding was anticipated because ADS051 contains a covalent 2000 MW PEG modification. The PEG solutions used to treat constipation and for colonic endoscopic preparation (3350 MW) also have minimal systemic exposure, with less than 0.25% of the relatively high doses administered to humans recovered in the urine [46]. In addition, the low cell permeability of ADS051 that we have demonstrated supports a lack of permeation across the GI epithelial mucosa, thereby limiting systemic exposure. Future studies in both healthy and diseased patients will be required to assess the impact of an inflamed GI tract on systemic exposure after oral dosing.

While ADS051 contains a CsA scaffold in its structure, it is not expected to significantly affect systemic immune cell function for several reasons. First, the covalently added linker was added at a position in the scaffold where modifications have been reported to significantly reduce the ability of the CsA to block calcineurin function [37, 38, 39]. Second, ADS051 has very low permeability in cell‐based assays, similar to Lucifer yellow. Thus, it cannot effectively engage intracellular immune‐modulating targets such as calcineurin because it is unable to enter cells. In support of this, ADS051 exhibited no T‐cell inhibition (Fig. 4) at 10 μm compared with CsA in cell‐based assays. In monkey studies, very low systemic exposure was observed at a potential clinically relevant oral dose of 50 mg·kg^−1^·day^−1^, with a whole blood *C*_max_ of less than 50 ng·mL^−1^. If this observation translates to humans, the low systemic exposure will likely further limit ADS051's effect on general host immunity after oral dosing.

ADS051 represents the first example of a rationally designed neutrophil modulator created through targeted medicinal chemistry that (a) eliminates the primary pharmacological activity of its parent (CsA‐mediated calcineurin/cyclophilin block) and (b) allows for its use at higher doses than CsA, such that the parent drug's secondary pharmacological activities (MRP2 and FPR1 pathway block) become safely exploitable. The findings from our *in vitro* efficacy experiments and preclinical pharmacology and toxicology studies support the clinical development of this oral, selective neutrophil activity modulator in neutrophil‐mediated diseases.

## Materials and methods

### Cell lines

ADS051 efficacy/permeability was evaluated utilizing *in vitro* assays with human‐derived colonic epithelial cells (T84 and Caco‐2) and neutrophils (human polymorphonuclear cells).

T84 intestinal epithelial cells at passages 50–79 (ATCC) were grown in growth media (Life Technologies Corporation, Grand Island, NY, USA; a 1 : 1 mixture of Dulbecco's modified Eagle's medium [DMEM] and Ham's F12 nutrient mixture supplemented with 14 mm NaHCO_3_, 15 mm HEPES buffer [N‐2‐hydroxyethylpiperazine‐N′‐2 ethanesulfonic acid], 100 units·mL^−1^ penicillin/streptomycin (pen/strep), and 5% heat‐inactivated fetal bovine serum [FBS; Cytiva, Logan, UT, USA]). Cells were maintained at 37 °C, 5% CO_2_. Monolayers were grown on collagen‐coated tissue culture‐treated 24‐well Transwell plates or 96‐well Transwell plates (Corning Costar, Corning, NY, USA) and used 6–8 days after plating.

Caco‐2 cells (ATCC) from a continuous culture were used to determine MRP2 and MDR1 inhibition. Cells at passage number 57 were seeded in 96‐well Transwell plates, cultured for 21–28 days in 115 μL Dulbecco's modified Eagle's medium (Life Technologies Corporation) supplemented with 1% l Glutamax, 1% pen/strep, and 10 mm HEPES, plus 10% FBS (Cytiva) and incubated at 37 °C, 5% CO_2_, in a humidified atmosphere.

As previously described, human polymorphonuclear cells (neutrophils) were isolated from whole blood obtained from normal healthy human volunteers by venipuncture [47] (IRB ID 13006_9; U Mass Chan Medical School). We can confirm that written informed consent has been obtained from each human subject and that all experiments with human samples conform to the Declaration of Helsinki. Specifically, Study ID: 13006; Fact Sheet version 3; 09/09/2013; Template v.11/15/12, entitled University of Massachusetts Medical School Committee for the Protection of Human Subjects in Research, included the name of the research study (Mucosal Inflammation Orchestrated by Pathogens), its purpose (utilizing blood from normal human volunteers to isolate neutrophils in order to investigate the effects of these cells on intestinal or lung cultured cell lines), and indicated that participation was voluntary.

Peripheral blood mononuclear cells was used in the T‐cell activation/inhibition assays (cryopreserved PBMCs from 3 human donors; Eurofins Panlabs, catalog # 960010).

### Neutrophil transmigration (chemotaxis) and activation assays

The assays, which recapitulate the *in vivo* human physiology of neutrophil‐driven colonic inflammation, were modified to assess the efficacy of ADS051. In the neutrophil transmigration assay (a modified Boyden chamber assay), a polarized T84 human intestinal cell line monolayer was grown on a Transwell insert to mimic the intestinal epithelial barrier. The apical side of the monolayer corresponded to the luminal space in the GI tract. The cells were treated with ADS051 at various concentrations on the apical side. ADS051 and probenecid (Sigma‐Aldrich, St. Louis, MO, USA) control (a commonly used MRP2 inhibitor [32]) were assessed in a concentration range of 32 nm to 1 μm (ADS051) and at 100 μm (probenecid). The cells were then apically stimulated by adding pathogenic *S. typhimurium*, eliciting HXA_3_ efflux via MRP2 into the apical side of the chamber. Fresh human neutrophils, added to the basolateral side of the monolayer, detected the HXA_3_ gradient and migrated through the epithelial monolayer to the apical side, where they were quantified by assay of MPO activity, a neutrophil biomarker [47].

In another version of the assay that used to determine neutrophil migration and activation by fMLF (Sigma‐Aldrich) stimulation, fresh human neutrophils were incubated with ADS051 or control media for 1 h at 37 °C and added to the basolateral side of an unstimulated intestinal cell monolayer system described above. A potent neutrophil chemo‐attractant and activator, fMLF, was added to the apical side of the Transwell to achieve a concentration of 100 nm. The fMLF‐mediated neutrophil migration and activation, mediated by the FPR1, were measured by assay of MPO activity.

### Efflux inhibition assays

The ADS051 inhibitory activity on the efflux of an MRP2 substrate, saquinavir (Sigma‐Aldrich), or an MDR1 substrate, talinolol (Toronto Research Chemicals, Toronto, ON, Canada), in a Caco‐2 monolayer system was investigated to evaluate ADS051 efficacy in an assay orthologous to the neutrophil transmigration studies described above. Caco‐2 cells (described earlier) were incubated with either 10 μm saquinavir alone, 10 μm saquinavir with 20 μm MK‐571 (control inhibitor; Sigma‐Aldrich), 10 μm talinolol alone, 10 μm talinolol with 100 μm haloperidol (Sigma‐Aldrich) or verapamil (Sigma‐Aldrich) 25 μm (control inhibitors), or 10 μm substrate (saquinavir or talinolol) with ADS051 at 25 μm, as shown in Fig. S1. The involvement of efflux transporters was evaluated based on bidirectional permeability. Samples from the apical and basolateral sides at time zero and after 2 h of incubation at 37 °C were analyzed by LC‐MS/MS. Calculations were performed using peak area ratios.

### Bioanalytical methods

The GLP‐compliant bioanalytical method development and validation studies conducted at Charles River Laboratories (Ashland, OH) to support ADS051 detection in whole blood and feces in Sprague–Dawley rats and cynomolgus monkeys employed an ultra‐high performance liquid chromatography–tandem mass spectrometry assay after solid phase extraction of the samples.

### Animal and ethics statement

The protocol and any amendment(s) or procedures involving the care and use of animals in this study were reviewed and approved by Institutional Animal Care and Use Committee (IACUC) at Charles River Laboratories (Shrewsbury, MA). During the study, the care and use of animals were conducted with guidance from the USA National Research Council guidelines. Veterinary care was available throughout the study for animals to be examined by the veterinary staff as warranted by clinical signs or other changes. There were no changes in the animals that required veterinary intervention to ameliorate pain or distress.

For the preclinical studies, young adult male and female Sprague–Dawley rats and cynomolgus monkeys were utilized in repeat oral dose toxicology and *in vivo* safety pharmacology studies at Charles River Laboratories. The monkey was chosen as the nonrodent species for the repeat dose toxicity testing because (a) its GI tract is physiologically and immunologically similar to humans, and (b) it is less prone to emesis after oral administration of test drugs than other nonrodent species (i.e., dogs). Therefore, it is a good model for studying the potential toxicological effects of orally administered ADS051. The IACUC protocol numbers for the rat and monkey 28‐day toxicology studies are 20177387 and 20177386, respectively. The protocol was approved by the Committee on the Ethics of Animal Experiments of the University of Minnesota (Protocol Number: 27‐2956).

For the rat studies, the housing setup was as specified in the USDA Animal Welfare Act (9 CFR, Parts 1, 2, and 3) and as described in the *Guide for the Care and Use of Laboratory Animals* (National Academy Press, 8th edition). Details can be found under Supporting Information.

For the monkey studies, the housing setup was as specified in the USDA Animal Welfare Act (9 CFR, Parts 1, 2, and 3) and as described in the *Guide for the Care and Use of Laboratory Animals* (National Academy Press, 8th edition). Details can be found under Supporting Information.

### *In vivo* safety pharmacology

*In vivo* safety pharmacology studies were conducted in Sprague–Dawley rat and cynomolgus monkey to assess potential ADS051‐mediated GI, neurological, respiratory (in rat), and cardiovascular (in cynomolgus monkey) off‐target effects. In these studies, ADS051 was administered orally. Postoral dosing with ADS051 was detected and quantified in plasma/whole blood and feces.

To evaluate the effects of ADS051 on GI motility when administered at 0, 50, 300, and 1000 mg·kg^−1^ as a single dose via oral gavage, six male Crl:CD(SD) Sprague–Dawley rats per group were used, with CD(SD) being the rat strain and Crl the substrain. Animals received a single dose of ADS051 via oral gavage. A charcoal suspension was dosed as a separate administration via oral gavage approximately 30 min following the test article or the vehicle dose. The test article and charcoal suspension dose volumes were 10 mL·kg^−1^. Thirty minutes after charcoal suspension administration by oral gavage, animals were sacrificed, their intestines removed, and intestinal charcoal transit distance (calculated percent distance traveled and normalized percent inhibition) was evaluated.

### Repeat‐dose toxicity

The GLP 28‐day, repeat oral toxicity studies were conducted in Sprague–Dawley rats and cynomolgus monkeys. ADS051 was formulated in calcium‐ and magnesium‐free phosphate buffered saline, 1X (pH 7.2; “vehicle”) for the 10 mL·kg^−1^ dose volume administration. The ADS051 single, oral dosages for these definitive GLP studies were 0 (vehicle only), 50, 300, and 1000 mg·kg^−1^·day^−1^. ADS051 was administered orally by gavage once daily for 28 days to three groups of 10 male and 10 female Sprague–Dawley rats, and a fourth group of 10 male and 10 female rats were dosed with the vehicle. Five additional male and female rats were included in the vehicle and high‐dose groups as recovery phase comparators (sacrificed at day 56 after 28 days of no test article or vehicle dosing). In the cynomolgus monkey study, ADS051 was administered orally by gavage for 28 days to three groups of three male and three female cynomolgus monkeys. A control group, consisting of three animals per sex, received vehicle for 28 days. Two additional males and females were included in the vehicle and high‐dose groups as recovery phase comparators (sacrificed at day 56 after 28 days of no dosing). The dose volume was 10 mL·kg^−1^ for all groups. The following parameters and end points were evaluated in these studies: clinical signs, body weights, body weight gain, ophthalmology (monkey only), ECGs (monkey only), clinical pathology parameters (hematology, coagulation, clinical chemistry, and urinalysis), TK assessment of ADS051 in whole blood, fecal concentrations of ADS051, gross necropsy findings, organ weights (organ‐to‐body weight and organ‐to‐brain weight ratios), and microscopic histopathologic examinations. On Days 1 and 28, TK whole blood samples were obtained predose and at 0.25, 0.5, 1, 2, 4, 6, 8, and 24 h postdose. Blood samples were collected from a jugular (rat) or inguinal (monkey) vein into tubes containing K_3_EDTA. Whole blood concentrations of ADS051 were measured using a validated analytical procedure. The results of these analyses were used for TK evaluation. Noncompartmental TK parameters were estimated using phoenix version 1.4 winnonlin software (version 6.4). A noncompartmental approach consistent with the oral gavage route of administration was used for the parameter estimation. ADS051 quantification was carried out in the homogenized fecal samples taken at 0 to 8 h and 8 to 24 h of postdose following the first day of dosing (Day 1) and on Day 22 for the rats and Day 24 for monkeys in the 28‐day studies.

### Statistical analyses

Statistical significance (*t*‐test or ANOVA, as specified in their respective graphs) was calculated, and graphs were constructed using graphpad prism version 9.0 (GraphPad Software, San Diego, California, USA).

### T‐cell inhibition assays

Pharmacodynamic analyses were performed to assess T‐cell immunomodulatory activity. Cryopreserved PBMCs (Eurofins Panlabs, catalog # 960010) from three healthy human donors were drip‐thawed. Cells were diluted to the appropriate density (1 × 10^5^ per well) and seeded into 96‐well polypropylene plates with culture medium (Roswell Park Memorial Institute Medium 1640, 10% heat‐inactivated FBS, 1% pen/strep, and 2 mm l‐glutamine, from Thermo Fisher Scientific). The cells were incubated at 37 °C, 5% CO_2_ for 1 h prior to the addition of test compounds or controls (CsA or culture medium alone). Next, test compounds or controls were added and incubated at 37 °C, 5% CO_2_ for 1 h. Anti‐CD3 antibody clone OKT3 was added, and the cultures were incubated at 37 °C, 5% CO_2_ for 24 and 48 h. Test samples were diluted with assay buffer. Each sample's cytokine/chemokine levels were determined using Luminex methodology as per the manufacturer's protocol, utilizing the MILLIPLEX Human Cytokine/Chemokine Magnetic Bead Panel from Millipore Sigma (catalog # HCYTOMAG‐60 K).

## Conflict of interest

The authors of this manuscript have the following competing interests: CKM is an employee of Adiso Therapeutics, Inc with stock options. CKM is an inventor of patents owned by Adiso Therapeutics, Inc. BD is an employee of Adiso Therapeutics, Inc. FBO is an independent nonclinical development consultant to Adiso Therapeutics, Inc and has received compensation for the consultancy with Bacainn Biotherapeutics Ltd, Artugen Therapeutics Ltd, ClearB Therapeutics Ltd, and Epsila Bio, Inc with no other financial disclosures to declare. RED has no financial disclosures to declare. RF received compensation salary from commercial companies. In the last 5 years, this has included Artugen Therapeutics Ltd, Bacainn Biotherapeutics Ltd, ClearB Therapeutics Ltd, and Epsila Bio, Inc. BAM is a coinventor on a patent (PGT/US 18/42116). She and her academic institution stand to gain financially through potential commercialization outcomes resulting from activities associated with the licensing of that intellectual property.

## Author contributions

CKM was involved in conceptualization, data curation, funding acquisition, investigation, methodology, project administration, supervision, validation, visualization, writing—original draft preparation, writing—review & editing. BD was involved in funding acquisition, investigation, methodology, resources, supervision, writing—review & editing. FBO was involved in methodology, analysis, writing—review & editing. RED was involved in conceptualization, methodology, supervision, writing—review & editing. RF was involved in conceptualization, funding acquisition, methodology, supervision, writing—review & editing. BAM was involved in conceptualization, methodology, project administration, supervision, writing—review & editing.

## Supporting information


**Fig. S1.** Efflux inhibition by ADS051.
**Fig. S2.** ADS051 does not inhibit cytokine secretion from activated human T cells (48 h).

## Data Availability

All relevant data are within the manuscript and its Supporting Information files. Additional data marked in the article as “not shown” will be shared upon request. Please contact Christopher K. Murphy at cmurphy@adisotx.com.
