# Supplementary material for: Development of ADS051, an oral, gut‐restricted, small molecule neutrophil modulator for the treatment of neutrophil‐mediated inflammatory diseases
Source: FEBS Open Bio. 2023 Jul 10;13(8):1434–46. doi: 10.1002/2211-5463.13668 (PMC10392058; doi:10.1002/2211-5463.13668)
Supplement: Supplementary file 1 — Fig. S1. Efflux inhibition by ADS051. Fig. S2. ADS051 does not inhibit cytokine secretion from activated human T cells (48 h). [file FEB4-13-1434-s001.docx]

**SUPPORTING INFORMATION**

**Title:**

*Development of ADS051, an oral, gut-restricted, small molecule as a neutrophil modulator for the treatment of neutrophil-mediated diseases*

**Authors:**

Christopher K. Murphy

Bharat Dixit

Frederick B. Oleson

Roland E. Dolle

Ronald Farquhar

Beth A. McCormick

**Materials Included:**

Supporting Information Figures and Legends: 2

**Figure S1:** Efflux inhibition by ADS051


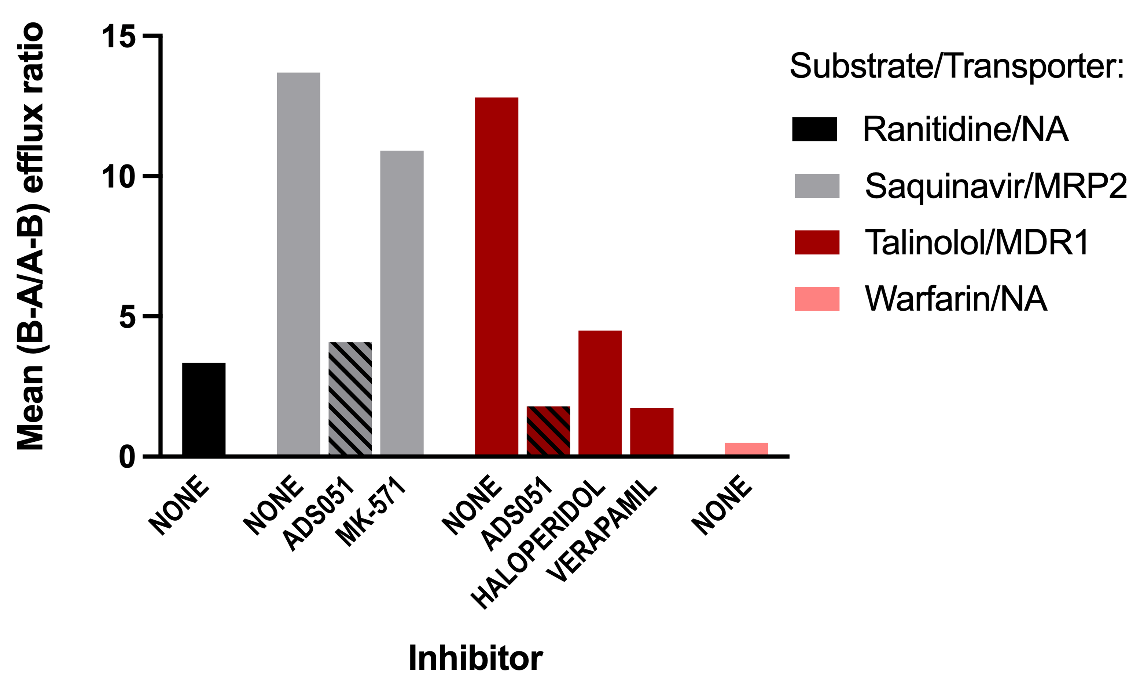


ADS051 was evaluated for inhibition of efflux activity using human-derived, colonic epithelial Caco2 cell monolayers expressing MRP2 or MDR1. The permeability of their respective substrates (saquinavir or talinolol) was determined following the addition of ADS051 or a control inhibitor. The efflux transport of the respective substrates was evaluated based on bidirectional permeability. The substrate concentrations in the apical and basolateral compartments at time zero and after 2 hours of incubation at 37 °C were analyzed by LC‑MS/MS. Calculations were performed using peak area ratios.

*Incubation Time (h): 2; Substrate Concentration (μM): 10; Inhibitor Concentration (μM): ADS051: 25; MK-571: 20; Haloperidol: 100; Verapamil: 25; (A): Substrate; (B): Transporter.*

*Permeability assessments (B-A, A-B) were performed in triplicate and the %CV did not exceed 25.*

**Figure S2:** ADS051 does not inhibit cytokine secretion from activated human Tcells (48 h)

**
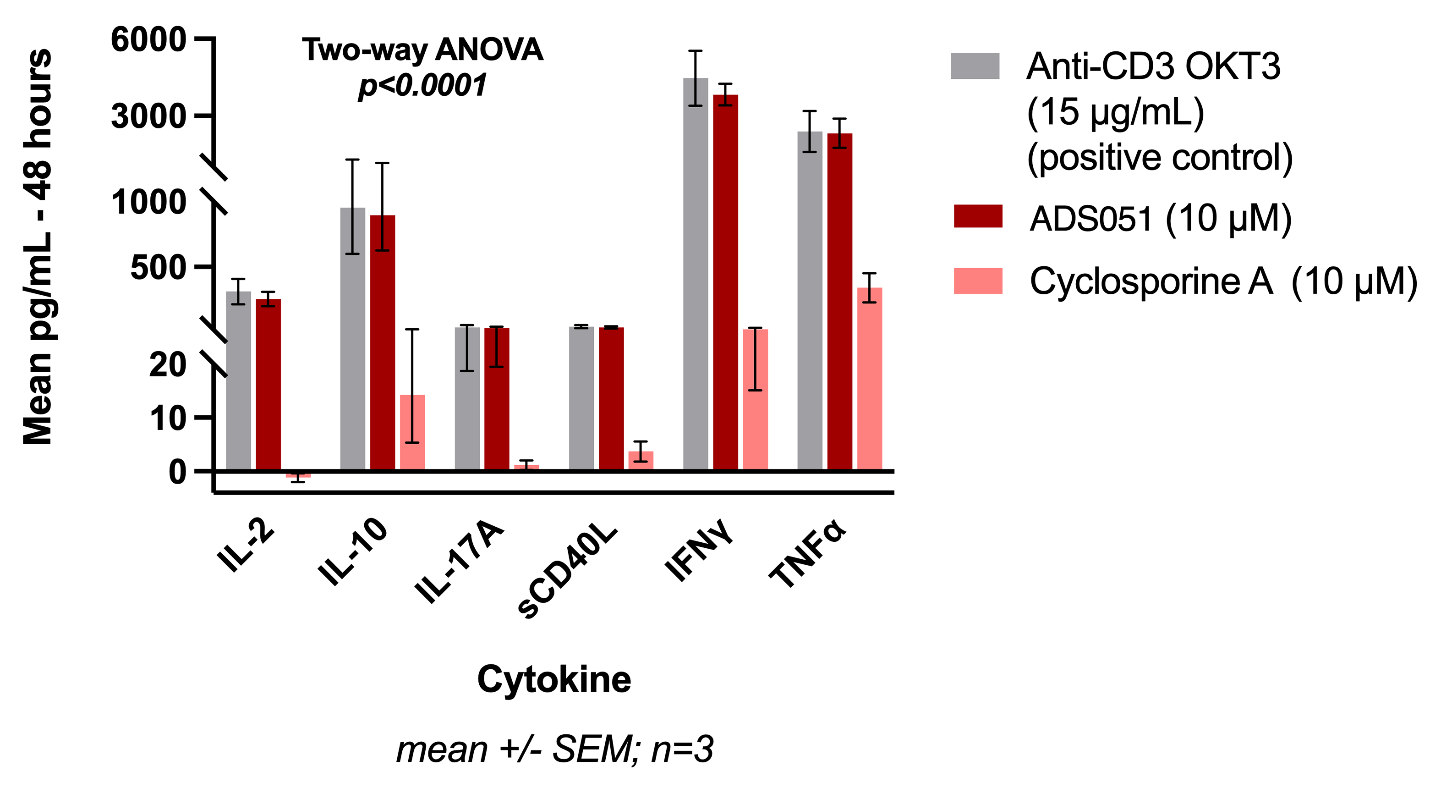
**

To assess T-cell immunomodulatory activity, T-cell activation/inhibition assays were performed using human peripheral blood mononuclear cells (PBMC) from 3 healthy human donors. The cells (1 x 10^5^ per well), seeded into 96-well plates, were incubated at 37° C, 5% CO_2,_ for 1 hour prior to the addition of test compounds or controls (CsA or culture medium alone). The test compounds or controls were added and incubated at 37 °C, 5% CO_2,_ for 1 hour. Anti-CD3 antibody clone OKT3 was added to initiate T-cell activation, and the cultures were incubated at 37 °C, 5% CO_2_ for 48 hours. The cytokine/chemokine levels in each sample were determined using Luminex methodology. Statistical analysis was performed using GraphPad Prism’s 2-way ANOVA test (*P* < .0001).

*(GraphPad Prism formatting style for p values: *P < .05, **P < .01, ***P < .001, ****P < .0001).*
